# Prevalence of hyponatremia among older inpatients in a general hospital

**DOI:** 10.1007/s41999-020-00320-3

**Published:** 2020-05-05

**Authors:** Xu Zhang, Xiao-Ying Li

**Affiliations:** 1 Medical School of Chinese PLA General Hospital, Beijing, China; 2 Department of Cardiology, The Second Medical Center of PLA General Hospital, 28 Fuxing Road, Haidian District, Beijing, 100853 China

**Keywords:** Hyponatremia, Older, Epidemiology

## Abstract

**Purpose:**

This study aimed to explore the incidence, clinical features, etiology, and mortality of hyponatremia in older inpatients and thus provide preliminary data for an epidemiological study.

**Methods:**

Hospitalized older patients diagnosed with hyponatremia at the First Medical Center of PLA General Hospital during January 2013–December 2016 were stratified by serum sodium concentrations into mild (130– < 135 mmol/L), moderate (125– < 130 mmol/L) and severe hyponatremia groups (< 125 mmol/L). Etiologies, medication histories, hospitalization times, and outcomes were analyzed.

**Results:**

During the indicated period, 4364 older patients with hyponatremia were hospitalized, including 2934 men and 1430 women with an average age of 84.6 ± 3.5 years (range 80–104 years). The prevalence of hyponatremia was 24.7%. An analysis of common primary diseases identified respiratory diseases as the most frequent (25.0%), followed by tumors (23.1%), cardiovascular diseases (19.9%), central nervous system diseases (8.9%), and orthopedic diseases (6.1%). PPIs (59.7%), loop diuretics (57.4%), potassium-preserving diuretics (29.5%), ACEIs/ARBs (20.0%), thiazide diuretics (12.5%), and NSAIDs (12.4%) were the drugs most commonly associated with hyponatremia. The in-hospital mortality rate was 11.7%. Aggravated hyponatremia led to a prolonged hospitalization time. Moreover, when compared with mild hyponatremia, moderate and severe hyponatremia were associated with significant increases in in-hospital mortality (ORs 1.89 and 2.66, respectively; 95% CIs 1.54–2.33 and 2.06–3.43, respectively; *P* < 0.01).

**Conclusions:**

Hyponatremia is a common complication in hospitalized older patients and is caused mainly by respiratory diseases, tumors, and cardiovascular diseases. Given the correlation between the degree of hyponatremia and prognosis, the early and accurate identification and treatment of this condition can reduce the associated morbidity and mortality.

## Key Summary Points

**Aim:**

This study aimed to explore the prevalence and the in-hospital mortality of hyponatremia in older inpatients who were diagnosed and treated at a single center.

**Findings:**

Nearly a quarter of older inpatients presented with hyponatremia, which was most frequently associated with common primary diseases such as respiratory diseases, tumors, cardiovascular diseases, central nervous system diseases, and orthopedic diseases. Patients were most likely to use drugs such as PPIs, loop diuretics, potassium-preserving diuretics, ACEIs/ARBs, thiazide diuretics and NSAIDs, which were often associated with hyponatremia. The in-hospital mortality rate among patients with hyponatremia was 11.7%, and this rate increased with the severity of the disorder.

**Message:**

Early mobilisation on the day of/after surgery should be added as a new formal hip fracture standard of care in keeping with best international practice.

## Introduction

Hyponatremia is a clinical condition characterized by a serum sodium concentration of < 135 mmol/L, regardless of changes in the extracellular fluid volume. This is the common electrolyte disorder observed in clinical settings [1–4], with incidences ranging from 15 to 30% among hospitalized patients [5]. Hyponatremia is particularly common among older people [6, 7]. Previously, a high incidence of hyponatremia in older patients was attributed mainly to the following factors: increased prevalence of comorbidities, frequent use of drugs that may lead to hyponatremia, age-related changes in homeostasis, and cognitive dysfunction [8].

It is recognized that severe hyponatraemia can be symptomatic and life threatening. In most cases, hyponatraemia is mild and asymptomatic; however, recent evidence shows that mild chronic ‘asymptomatic’ hyponatraemia, particularly in an older population, may contribute to impaired cognition [9], increased risk of falls and fractures [10, 11]. Winzeler et al. found that serum sodium was positively correlated with mortality, and serum sodium < 125 mmol/L was significantly correlated with 1-year mortality, recurrence rate of hyponatremia, and readmission rate [12]. Gomez-Hoyos et al. found that hyponatremia is associated with increased mortality and morbidity [13]. However, there is still some debate over how much of the increased risk of death is directly attributable to hyponatraemia, and how much is due to under-lying disease.

Considering the aging of the population, the increased susceptibility to electrolyte disturbances and the increased incidence rate associated with hyponatremia in old people, hyponatremia is of increasing importance. At present, there is no study providing the frequency of hyponatremia in older inpatients for general medical conditions in China. In our study, we conducted a retrospective analysis of hyponatremia in older inpatients (age ≥ 80 years) in a general hospital (the First Medical Center of PLA General Hospital). These analyses encompassed community-acquired hyponatremia (CAH) and hospital-acquired hyponatremia (HAH). The purpose of our study is to explore the prevalence, mortality, etiology, disease characteristics, and prognosis of hyponatremia in older inpatients, so as to provide more valuable reference for clinical medical workers.

## Methods

Our study is a descriptive and retrospective analysis of inpatient medical records. The First Medical Center of PLA General Hospital, which is a 3000 bed tertiary teaching hospital and 165 clinical and medical technology departments, provides care to a economically diverse population within the northern part of China and the surrounding region. We searched the laboratory database of inpatients in the hospital to identify all patients with hyponatremia (serum sodium < 135 mmol/L). We analyzed the serum sodium level of all patients, and corrected the serum sodium value of the patients with hyperglycemia or hyperlipidemia before entering the group [14], so as to exclude the patients with pseudohyponatremia.

From January 2013 to December 2016, 17,693 older patients (age ≥ 80 years) were hospitalized at the First Medical Center of PLA General Hospital. All patients underwent a serum electrolyte analysis, and hyponatremia was diagnosed in patients with a serum sodium concentration of < 135 mmol/L. During the indicated time period, 4364 older patients with hyponatremia were hospitalized at our institution, including 2934 men and 1430 women with an average age of 84.6 ± 3.5 years (range 80–104 years). These patients were subsequently classified according to the serum sodium concentration at admission into mild (≥ 130–< 135 mmol/L), moderate (≥ 125–< 130 mmol/L) and severe hyponatremia groups (< 125 mmol/L). On further analysis of patients with hyponatremia, those with blood sodium < 135 mmol/L within 24 h after admission were defined as having CAH, and the patients with blood sodium < 135 mmol/L within 24 h after admission were defined as having HAH.

For all patients, the age, sex, body mass index, hospitalization time, minimum blood sodium concentration, symptoms related to hyponatremia, history of primary diseases and medication use, diagnosis and prognosis were recorded. Prognoses were classified as a cure, improvement, unhealed or death. Informed consent was provided by all patients. The study protocol was reviewed and approved by the Ethics Committee of The First Medical Center of PLA General Hospital.

The results of univariate analyses are expressed as means ± SDs. Independent sample *t* tests and chi-square tests were used for bivariate analyses, while an analysis of variance (ANOVA) was used for all comparisons involving more than two groups. A *P* value < 0.05 was considered statistically significant. Data were analyzed using SPSS 19.0 statistical software (IBM Corp., Armonk, NY, USA).

## Results

### Patient characteristics

The characteristics of the included patients are presented in Table 1. In our study, the overall prevalence of hyponatremia among older hospitalized patients admitted during the study period was 24.7% (4364/17,693). Among patients with hyponatremia, 2882 cases (66.0%) were classified as mild hyponatremia, of which 1958 (67.9%) and 924 cases (32.1%) involved men and women, respectively. The overall prevalence of mild hyponatremia was 16.3% (2882/17,693). Additionally, 1037 cases (23.8%) were classified as moderate hyponatremia, of which 695 (67.0%) and 342 cases (33.0%) involved men and women, respectively. The overall prevalence of moderate hyponatremia was 5.9% (1037/17,693). A further 445 cases (10.2%) were classified as severe hyponatremia, of which 281 (63.2%) and 164 cases (36.9%) involved men and women, respectively. The overall prevalence of severe hyponatremia was 2.5% (445/17,693). The groups did not differ significantly with respect to the sex distribution.

**Table 1:** General clinical data of each group of hospitalised older patients

	Mild hyponatremia group (*N* = 2882)	Moderate hyponatremia group (*N* = 1037)	Severe hyponatremia group (*N* = 445)	Total (*N* = 4364)
Male [*n* (%)]	1958 (67.9)	695 (67.0)	281 (63.2)	2934 (67.2)
Female [*n* (%)]	924 (32.1)	342 (33.0)	164 (36.9)	1430 (32.8)
Age, years (mean ± SD)	84.4 ± 3.5	84.9 ± 3.7*	84.8 ± 3.5*	84.6 ± 3.5
BMI, kg/m^2^ (mean ± SD)	22.4 ± 4.8	22.1 ± 4.1	21.5 ± 4.2*^#^	22.2 ± 4.6
Blood sodium level, mmol/L (mean ± SD)	132.8 ± 1.4	127.8 ± 1.4*	120.9 ± 4.1*^#^	130.4 ± 4.3
Hyponatremia subtypes				
CAH [*n* (%)]	1220 (42.3)	594 (57.3)*	283 (63.6)*^#^	2097 (48.1)
HAH [*n* (%)]	1662 (57.7)	443 (42.7)*	162 (36.4)*^#^	2267 (51.9)
Classification				
Hypovolemia [*n* (%)]	308 (10.9)	133 (13.1)	62 (13.9)	503 (11.7)
Euvolemia [*n* (%)]	1788 (63.4)	635 (62.3)	271 (60.9)	2694 (62.9)
Hypervolemia [*n* (%)]	724 (25.7)	251 (24.6)	112 (25.2)	1087 (25.4)
Others [*n* (%)]	62 (2.2)	18 (1.7)	0 (0.0)	80 (1.8)
Hospital outcome				
Hospitalisation time, days (mean ± SD)	25.2 ± 43.2	39.3 ± 70.8*	50.4 ± 78.8*^#^	31.1 ± 56.0
Cure [*n* (%)]	610 (21.2)	167 (16.1)*	68 (15.3)*	845 (19.4)
Improvement [*n* (%)]	1984 (68.8)	695 (67.0)	277 (62.3)*	2956 (67.7)
Unhealed [*n* (%)]	31 (1.1)	13 (1.3)	8 (1.8)	52 (1.2)
Death [*n* (%)]	257 (8.9)	162 (15.6)*	92 (20.7)*^#^	511 (11.7)

*BMI* body mass index, *SD* standard deviation, *CAH* community-acquired hyponatremia, *HAH* hospital-acquired hyponatremia

^*^P < 0.05, comparison with mild hyponatremia group

^#^*P* < 0.05, comparison with moderate hyponatremia group

Compared to the mild hyponatremia group, the moderate hyponatremia group had a significantly higher age, while the severe hyponatremia group had a significantly higher age and BMI. The severe hyponatremia group also had a significantly higher BMI than the moderate hyponatremia group. As expected, the blood sodium level decreased gradually with the increasing exacerbation of hyponatremia (Table 1).

According to the clinical manifestations of patients with hyponatremia (such as blood pressure, pulse, urine output, skin elasticity, color and temperature changes), we divided them into three subtypes (hypovolemic, euvolemic, and hypervolemic). Euvolemic hyponatremia was the most common subtype in each group; however, there was no significant difference among the subtypes (Table 1).

In our study, 2097 patients had CAH; the prevalence was 11.9% (2097/17,693), 2267 patients had HAH and the prevalence was 12.8% (2267/17,693). With the aggravation of hyponatremia, the proportion of CAH increased and the proportion of HAH decreased (Table 1).

### Distribution of primary diseases in each group

The results of an analysis of primary diseases is presented in Table 2. In our study, respiratory diseases were the most common primary diseases reported in older patients with hyponatremia (25.0%), followed by tumors (23.1%), cardiovascular diseases (19.9%), central nervous system diseases (8.9%), and orthopedic diseases (6.1%). In our study, pulmonary infection accounted for nearly all cases of respiratory disease (90.9%), lung cancer was the most common type of tumor (23.2%), heart failure accounted for more than three quarters of cardiovascular disease cases (81.7%), while cerebral infarction accounted for more than half of central nervous system diseases (52.2%) and fracture was the most common in orthopedic diseases (84.6%).

**Table 2:** Distribution of basic diseases in each group of hospitalised older patients [*n* (%)]

	Mild hyponatremia group (*N* = 2882)	Moderate hyponatremia group (*N* = 1037)	Severe hyponatremia group (*N* = 445)	Total (*N* = 4364)
Respiratory diseases	732 (25.4)	272 (26.2)	87 (19.6)*#	1091 (25.0)
Tumours	651 (22.6)	230 (22.2)	128 (28.8)*#	1009 (23.1)
Cardiovascular diseases	593 (20.6)	198 (19.1)	77 (17.3)	868 (19.9)
Central nervous system diseases	196 (6.8)	118 (11.4)*	73 (16.4)*#	387 (8.9)
Kidney diseases	110 (3.8)	49 (4.7)	9 (2.0)^#^	168 (3.9)
Digestive diseases	70 (2.4)	29 (2.8)	26 (5.8)*^#^	125 (2.9)
Endocrine diseases	31 (1.1)	11 (1.1)	2 (0.5)	44 (1.0)
Hepatobiliary diseases	180 (6.3)	43 (4.2)*	15 (3.4)*	238 (5.5)
General surgical diseases	42 (1.5)	17 (1.6)	6 (1.4)	65 (1.5)
Urological diseases	37 (1.3)	14 (1.4)	4 (0.9)	55 (1.3)
Orthopaedic diseases	203 (7.0)	45 (4.3)*	18 (4.0)*	266 (6.1)
Others	37 (1.3)	11 (1.1)	0 (0.0)	48 (1.1)

^*^P < 0.05, comparison with mild hyponatremia group

^#^*P* < 0.05, comparison with moderate hyponatremia group

We considered the diagnosis of syndrome of inappropriate antidiuretic hormone secretion (SIADH) based on five criteria [15]:

1. Decreased effective osmolality of the extracellular fluid (Posm < 275 mOsmol/kg H_2_O).
2. Inappropriate urinary concentration (Uosm > 100 mOsmol/kg H_2_O with normal renal function) at some level of plasma hypo-osmolality.
3. Clinical euvolemia, as defined by the absence of signs of hypovolemia (orthostasis, tachycardia, decreased skin turgor, dry mucous membranes) or hypervolemia (subcutaneous edema, ascites).
4. Elevated urinary sodium excretion (> 20–30 mmol/L) under normal salt and water intake.
5. Absence of other potential causes of euvolemic hypo-osmolality: severe hypothyroidism, hypocortisolism.

Among the patients with hyponatremia, 1240 patients (28.4%) met the diagnostic criteria of SIADH. The most common causes of SIADH were respiratory diseases (*n* = 731, 59.0%), followed by malignant tumors (*n* = 357, 28.8%) and central nervous system diseases (*n* = 128, 10.3%).

### Clinical application of drugs in each group

We counted the drugs used during hospitalization in patients, which were often associated with hyponatremia. As shown in Table 3, PPIs (59.7%), loop diuretics (57.4%), potassium-preserving diuretics (29.5%), ACEIs/ARBs (20.0%), thiazide diuretics (12.5%), and NSAIDs (12.4%) were the drugs most commonly administered. The frequencies of use of loop diuretics, thiazide diuretics, potassium-preserving diuretics, and PPIs were significantly higher in the moderate hyponatremia group than in the mild hyponatremia group. Moreover, the frequencies of use of loop diuretics, thiazide diuretics, potassium-preserving diuretics, mannitol, PPIs, ACEIs/ARBs, and NSAIDs were significantly higher in the severe hyponatremia group than in the mild hyponatremia group. The frequencies of use of loop diuretics and thiazide diuretics were significantly higher in the severe group than in the moderate group.

**Table 3:** Clinical applications of drugs in each group of hospitalised older patients [*n* (%)]

	Mild hyponatremia group (*N* = 2882)	Moderate hyponatremia group (*N* = 1037)	Severe hyponatremia group (*N* = 445)	Total (*N* = 4364)
Loop diuretics	1539 (53.4)	648 (62.5)*	316 (71.0)*^#^	2503 (57.4)
Thiazide diuretics	300 (10.4)	155 (15.0)*	91 (20.5)*^#^	546 (12.5)
Potassium-preserving diuretics	772 (26.8)	344 (33.2)*	169 (38.0)*	1285 (29.5)
Mannitol	56 (1.9)	28 (2.7)	16 (3.6)*	100 (2.3)
PPIs	1652 (57.3)	661 (63.7)*	292 (65.6)*	2605 (59.7)
ACEIs/ARBs	550 (19.1)	220 (21.2)	103 (23.2)*	873 (20.0)
SSRIs	52 (1.8)	25 (2.4)	12 (2.7)	89 (2.0)
Carbamazepine	27 (0.9)	11 (1.1)	5 (1.1)	43 (1.0)
NSAIDs	342 (11.9)	129 (12.4)	70 (15.7)*	541 (12.4)

*PPIs* proton pump inhibitors, *ACEIs/ARBs* angiotensin converting enzyme inhibitors/angiotensin II receptor blockers, *SSRIs* selective serotonin reuptake inhibitors, *NSAIDs* non-steroidal anti-inflammatory drugs

^*^P < 0.05, comparison with mild hyponatremia group

^#^*P* < 0.05, comparison with moderate hyponatremia group

### Prognostic analysis of each group

The results of the prognostic analysis are presented in Table 1. In our study, the in-hospital mortality of patients with hyponatremia was 11.7%, and that of the mild, moderate, and severe group was 8.9%, 15.6%, and 20.7%, respectively. With the aggravation of hyponatremia, the hospitalization time and mortality of each group were prolonged. Moreover, when compared with mild hyponatremia, moderate, and severe hyponatremia were associated with significantly increased in-hospital mortality (ORs 1.89 and 2.66, respectively; 95% CIs 1.54–2.33 and 2.06–3.43, respectively; *P* < 0.01). Compared with the moderate hyponatremia group, severe hyponatremia also significantly increased the in-hospital mortality (ORs 1.41, 95% CIs 1.06–1.87, *P* < 0.05).

In our study, the primary diseases of patients with hyponatremia were respiratory system diseases, tumors, cardiovascular system diseases, central nervous system diseases, and orthopedic diseases. The in-hospital mortality of the above diseases according to the specific disease name are pulmonary infection 15.0%, lung cancer 20.5%, heart failure 11.7%, cerebral infarction 16.3%, fracture 2.7%.

## Discussion

Hyponatremia is a common electrolyte disorder observed in clinical settings [1–4], particularly in critically ill and older patients. It is associated with poor clinical outcomes such as increased mortality, disability, prolonged hospital stay, and increased hospital costs [16–18]. Published data show that even mild and chronic hyponatraemia represents an economic burden [17, 18].

Previous studies on older patients have reported a prevalence of chronic hyponatremia of 8% among those living in private homes [19]. Tay et al. measured the electrolytes of 5873 outpatients (age ≥ 60 years) and found that 403 (6.9%) had hyponatremia in at least one blood test [20]. In a study of 118 residents (mean age 85.5 years) in a single geriatric health service facility, Naka et al. reported that 33 patients (28%) had hyponatremia [21]. In a retrospective study of inpatients at a tertiary hospital, Amit et al. reported that the incidence of hyponatremia in older patients (average age 73.9 ± 6.5 years) was 26.0% [22]. In a study of 8377 emergency patients, Lmai et al. reported a significantly higher prevalence of hyponatremia among older patients (≥ 65 years old, 17.0%) than among younger adults (18–64 years, 5.7%). Moreover, in that study, the prevalence of severe hyponatremia (serum sodium < 125 mmol/L) was significantly higher among older patients relative to younger adults (1.9% vs. 0.3%) [23]. In a comparative study of 2849 older patients (≥ 60 years) and 6756 non-older patients (13–59 years) at a single center during the same period, Siregar reported that the incidence of hyponatremia in older inpatients in a general ward setting was 2.43 times that observed in non-older patients (26.32% vs. 10.85%), while the incidence of HAH in older inpatients was 2.79 times that reported in non-older patients (7.35% vs. 2.64%) [24].

In our study, the overall prevalence of hyponatremia among older hospitalized patients admitted to a single center within a 4-year period was 24.7%. Most cases involved mild hyponatremia, in contrast to similar previous studies. This difference may be attributable to differences in the criteria used to define hyponatremia and the population selection protocol, which fully explained the high incidence of hyponatremia in older hospitalized patients. Among the 4364 hospitalized patients with hyponatremia included in this study, those with moderate and severe hyponatremia had a significantly higher age than those with mild hyponatremia, indicating that increasing age is associated with a gradual degeneration of the tissues and organs of the body, impairment of the mechanisms that maintain normal body fluid homeostasis, and increased vulnerability to hyponatremia-related diseases and drugs. Accordingly, severe hyponatremia was more likely to occur with age.

In this study, 2097 patients (48.1%) had CAH on admission and 2267 patients (51.9%) had HAH after admission. The proportion of CAH increased with the severity of hyponatraemia. As previously reported, severe hyponatremia was more frequently community-acquired than hospital-acquired [2, 25]. We speculate that this may be related to more active serum sodium monitoring and related treatment after admission.

Many diseases have been associated with the onset of hyponatremia. It has been reported that pneumonia, heart failure, liver cirrhosis, malignant tumors, stroke, and other diseases are all prone to hyponatremia [26–30]. In our study, we analyzed the incidence of primary diseases in 4364 older patients with hyponatremia and identified respiratory diseases, tumors, cardiovascular diseases, central nervous system diseases, and orthopedic diseases as the most common, among which pulmonary infection, lung cancer, heart failure, cerebral infarction, and fracture accounted for the highest proportion. Most of the patients in our study had clinical euvolemia, which was likely partially attributable to the large number of patients (*n* = 1240) who met criteria for SIADH. In our study, SIADH was the most common cause of hyponatremia, accounting for 28.4%, which was similar to 31% reported by Amit et al. [22]. Further research showed that the proportion of respiratory diseases is the highest in those with SIADH, followed by malignant tumors and central nervous system diseases. Our results for SIADH were similar to a previous study [31]. Our findings indicate that the common causes of hyponatremia are respiratory system disease, tumor, cardiovascular system disease, central nervous system disease, etc. These diseases should be monitored carefully in older patients, and blood sodium concentrations should be monitored closely in clinical settings to ensure the earliest possible detection and correction of hyponatremia.

Many drugs used in clinical settings can also aggravate existing hyponatremia or induce iatrogenic hyponatremia. Several studies have reported that hyponatremia is caused by iatrogenic factors in 40–75% of patients [15]. The mechanisms associated with drug-induced hyponatremia include volume clearance, the effects of sodium ion reabsorption in renal tubules and the stimulation of abnormal antidiuretic hormone (ADH) secretion or enhancement of its activity. Consistent with our findings, previous studies have identified several drugs associated with the development of hyponatremia, including diuretics (especially thiazide diuretics), dehydrants, PPIs, ACEIs/ARBs, SSRIs, carbamazepine and NSAIDs [32]. In one report, 73%, 20% and 8% of cases of diuretic-related hyponatremia were induced by thiazide diuretics alone, a combination of thiazide and potassium-preserving diuretics or furosemide, respectively [33]. Another study reported that 13.7% of 951 thiazide-treated outpatients had hyponatremia and that age > 70 years was associated with a 3.9 times increase in hyponatremia risk [34]. Concomitant administration of drugs that affect water homeostasis, such as SSRIs, SNRIs, NSAIDs, or benzodiazepines, is frequent in older patients with thiazide-associated hyponatremia [5]. In a retrospective study of 2569 patients (aged > 65 years) in community-dwelling older people, Grattagliano et al. reported the likelihood of hyponatremia trebled when four drugs were taken, and it was seven-fold higher with the use of six drugs [19]. As mentioned earlier, our study identified PPIs, loop diuretics, potassium sparing diuretics, ACEIs/ARBs, thiazide diuretics and NSAIDs as drugs frequently associated with hyponatremia in patients. We further demonstrated that increasing hyponatremia severity was significantly associated with a higher frequency of loop diuretic and thiazide diuretic use, suggesting that these drugs are an important cause of hyponatremia in older patients. Moreover, we determined that the use of potassium-preserving diuretics and PPIs increased the incidence of moderate to severe hyponatremia and that the use of mannitol, ACEIs/ARBs, and NSAIDs increased the incidence of severe hyponatremia. These findings suggest the serum sodium concentrations of patients using these drugs in clinical settings should be monitored continuously, and the sodium intake should be increased appropriately as needed.

Early studies confirmed that hyponatremia is the most likely cause of deterioration and increased mortality associated with various internal environmental disorders [12, 13, 35, 36]. In a study of 53,236 hospitalized patients, Wald et al. noted reduced survival rates and prolonged hospitalization times among patients with CAH and HAH [35]. Particularly, in patients with serum sodium concentrations < 138 mmol/L, the mortality risks associated with CAH and HAH increased by 8% and 23%, respectively, with each 1-mmol/L decrease in the serum sodium concentration. Tzoulis et al. found that patients with hyponatremia and a serum sodium concentration < 128 mmol/L had a mortality rate of 17.3% and a risk of death during hospitalization 3.3 times higher than that of patients with a serum sodium concentration > 128 mmol/L [36]. Those findings suggest that hyponatremia is both an independent predictor of mortality and a direct cause of increased mortality. Consistent with those earlier findings, our analysis of older inpatients with different degrees of hyponatremia revealed that increasing severity was associated significantly with increases in the hospitalization time and mortality. This conclusion was reached by other scholars [1, 31]; however, it is at odds with the findings of Holland-Bill et al.[37]. In that study, the risk of mortality increased with the decrease in serum sodium, but the decrease in serum sodium below a threshold of 132 mmol/L did not contribute to a further increase in overall mortality risk. Further, our results are in disagreement with the findings of the study by Zheng et al.[38]. In this study, the in-hospital mortality was 1.7%, 11.1%, and 6.8% in the mild (≥ 130–< 135 mmol/L), moderate (≥ 125–< 130 mmol/L), and severe hyponatremia groups (< 125 mmol/L), respectively. Patients with moderate or severe hyponatremia had a higher mortality than those with mild hyponatremia (moderate vs. mild group: OR 6.92, 95% CI 2.53–18.92, *P* < 0.001; severe vs. mild group: OR 4.54, 95% CI 1.05–19.58, *P* = 0.043). There was no significant difference in the risk of death between the severe and moderate groups. In addition, in the specific analysis of primary diseases, the in-hospital mortality of patients with hyponatremia was higher in those with lung cancer, cerebral infarction and pulmonary infection (20.5%, 16.3%, 15.0%, respectively). The in-hospital mortality of heart failure patients was 11.7%, which was slightly lower than the above three diseases. We speculate that this may be related to the severity of primary diseases and the number of complications.

This study has several strengths. We studied a large and unselected population. As far as we know, this is the first study on the prevalence of hyponatremia in older inpatients in China. Our large research population enabled us to examine mortality risks associated with different levels of hyponatremia. However, our study has certain limitations. First, as this was a single center study with a relatively limited sample size, it was difficult to make precise clinico-etiological profiles and their association with different characteristics. Second, there was no data on post discharge mortality; this restricted the evaluation of in-hospital mortality, and therefore, we could not follow up on patients to assess long-term impact. Third, we did not have information regarding the use of outpatient drugs that were often associated with hyponatremia. Data including these drugs would enhance the information provided in our study. Fourth, The single serum sodium measurement in our study did not reflect changes in clinical management.

## Conclusion

Hyponatremia is a common electrolyte disorder observed in older inpatients. The severity of hyponatremia is related to in-hospital mortality. This disorder plays an important prognostic role and should receive careful clinical attention. Early prevention, detection, diagnosis and treatment strategies are needed to reduce the incidence and severity of hyponatremia, decrease the associated hospitalization times and mortality risks and improve the quality of life of older patients.

## Abbreviations

ACEIs/ARBs: Angiotensin-converting enzyme inhibitor/angiotensin II receptor blocker
ADH: Abnormal antidiuretic hormone
BMI: Body mass index
CAH: Community-acquired hyponatremia
CI: Confidence interval
HAH: Hospital-acquired hyponatremia
NSAIDs: Non-steroidal anti-inflammatory drugs
OR: Odds ratio
PPIs: Proton pump inhibitor
SD: Standard deviation
SIADH: Syndrome of inappropriate antidiuretic hormone secretion
SNRIs: Serotonin-norepinephrine re-uptake inhibitors
SSRIs: Selective serotonin reuptake inhibitors

## References

[1] Al Mawed S, Pankratz VS, Chong K (2018). Low serum sodium levels at hospital admission: outcomes among 2.3 million hospitalized patients. PLoS One.

[2] Krummel T, Prinz E, Metten MA (2016). Prognosis of patients with severe hyponatraemia is related not only to hyponatraemia but also to comorbidities and to medical management: results of an observational retrospective study. BMC Nephrol.

[3] Ingles Garces AH, Ang JE, Ameratunga M (2018). A study of 1088 consecutive cases of electrolyte abnormalities in oncology phase I trials. Eur J Cancer.

[4] Hao J, Li Y, Zhang X (2017). The prevalence and mortality of hyponatremia is seriously underestimated in Chinese general medical patients: an observational retrospective study. BMC Nephrol.

[5] Upadhyay A, Jaber BL, Madias NE (2006). Incidence and prevalence of hyponatremia. Am J Med.

[6] Liamis G, Filippatos TD, Elisaf MS (2016). Thiazide-associated hyponatremia in the elderly: what the clinician needs to know. J Geriatr Cardiol.

[7] Lindner G, Pfortmuller CA, Leichtle AB (2014). Age-related variety in electrolyte levels and prevalence of dysnatremias and dyskalemias in patients presenting to the emergency department. Gerontology.

[8] Soiza RL, Talbot HS (2011). Management of hyponatraemia in older people: old threats and new opportunitie. Ther Adv Drug Saf.

[9] Nowak KL, Yaffe K, Orwoll ES (2018). Serum sodium and cognition in older community-dwelling men. Clin J Am Soc Nephrol.

[10] Boyer S, Gayot C, Bimou C (2019). Prevalence of mild hyponatremia and its association with falls in older adults admitted to an emergency geriatric medicine unit (the MUPA unit). BMC Geriatr.

[11] Corona G, Norello D, Parenti G (2018). Hyponatremia, falls and bone fractures: a systematic review and meta-analysis. Clin Endocrinol (Oxf).

[12] Winzeler B, Jeanloz N, Nigro N (2016). Long-term outcome of profound hyponatremia: a prospective 12 months follow-up study. Eur J Endocrinol.

[13] Gomez-Hoyos E, Cuesta M, Del Prado-Gonzalez N (2017). Prevalence of hyponatremia and its associated morbimortality in hospitalized patients receiving parenteral nutrition. Ann Nutr Metab.

[14] Ganguli A, Mascarenhas RC, Jamshed N (2015). Hyponatremia: incidence, risk factors, and consequences in the elderly in a home-based primary care program. Clin Nephrol.

[15] Verbalis JG, Goldsmith SR, Greenberg A (2013). Diagnosis, evaluation, and treatment of hyponatremia: expert panel recommendations. Am J Med.

[16] Moreira DM, Gershman B, Lohse CM (2016). Paraneoplastic syndromes are associated with adverse prognosis among patients with renal cell carcinoma undergoing nephrectomy. World J Urol.

[17] Althaus AE, Krapf R (2018). Quality and cost considerations in hyponatraemic patients needing hospitalisation. Swiss Med Wkly.

[18] Corona G, Giuliani C, Parenti G (2016). The economic burden of hyponatremia: systematic review and meta-analysis. Am J Med.

[19] Grattagliano I, Mastronuzzi T, D'Ambrosio G (2018). Hyponatremia associated with long-term medication use in the elderly: an analysis in general practice. J Prim Health Care.

[20] Tay CL, Myint PK, Mohazmi M (2019). Prevalence and documented causes of hyponatraemia among geriatric patients attending a primary care clinic. Med J Malaysia.

[21] Naka T, Kohagura K, Kochi M (2018). Hyponatremia and mortality among very elderly residents in a geriatric health service facility. Clin Exp Nephrol.

[22] Jain AK, Nandy P (2019). Clinico-etiological profile of hyponatremia among elderly age group patients in a tertiary care hospital in Sikkim. J Family Med Prim Care.

[23] Lmai N, Osako K, Kaneshiro N (2018). Seasonal prevalence of hyponatremia in the emergency department: impact of age. BMC Emerg Med.

[24] Siregar P (2011). The risk of hyponatremia in the elderly compared with younger in the hospital inpatient and outpatient. Acta Med Indones.

[25] Hoorn EJ, Lindemans J, Zietse R (2006). Development of severe hyponatraemia in hospitalized patients: treatment-related risk factors and inadequate management. Nephrol Dial Transplant.

[26] Karki L, Thapa B, Sah MK (2016). Hyponatremia in patients with community acquired pneumonia. JNMA J Nepal Med Assoc.

[27] Shah V, Jahan N (2019). Prognostic significance of hyponatremia in ST-elevation myocardial infarction/heart failure patients. Cureus.

[28] Attar B (2019). Approach to hyponatremia in cirrhosis. Clin Liver Dis (Hoboken).

[29] Yoon J, Ahn SH, Lee YJ (2015). Hyponatremia as an independent prognostic factor in patients with terminal cancer. Support Care Cancer.

[30] Liamis G, Barkas F, Megapanou E (2019). Hyponatremia in acute stroke patients: pathophysiology, clinical significance, and management options. Eur Neurol.

[31] Gang X, Zhang Y, Pan X (2018). Hyponatremia: prevalence and characteristics in internal medicine patients in southeast of China. Medicine (Baltimore).

[32] Filippatos TD, Makri A, Elisaf MS (2017). Hyponatremia in the elderly: challenges and solutions. Clin Interv Aging.

[33] Hix JK, Silver S, Sterns RH (2011). Diuretic-associated hyponatremia. Semin Nephrol.

[34] Clayton JA, Rodgers S, Blakey J (2006). Thiazide diuretic prescription and electrolyte abnormalities in primary care. Br J Clin Pharmacol.

[35] Wald R, Jaber BL, Price LL (2010). Impact of hospital-associated hyponatremia on selected outcomes. Arch Intern Med.

[36] Tzoulis P, Bagkeris E, Bouloux PM (2014). A case-control study of hyponatraemia as an independent risk factor for inpatient mortality. Clin Endocrinol (Oxf).

[37] Holland-Bill L, Christiansen CF, Heide-Jørgensen U (2015). Hyponatremia and mortality risk: a Danish cohort study of 279 508 acutely hospitalized patients. Eur J Endocrinol Eur Feder Endocrine Soc.

[38] Zheng Y, Zheng FP, Li H (2020). The prevalence and causes of hyponatremia in hospitalized patients. Zhonghua Nei Ke Za Zhi.

